# Publishing quality improvement studies: learning to share and sharing to learn

**DOI:** 10.1016/j.bjao.2023.100123

**Published:** 2023-02-03

**Authors:** Duncan Wagstaff, Suneetha Ramani Moonesinghe

**Affiliations:** 1 Centre for Perioperative Medicine and Division of Surgery and Interventional Science, University College London, London, UK; 2 Department for Targeted Intervention, Division of Surgery and Interventional Science, University College London, London, UK

**Keywords:** Quality improvement, Improvement science, Perioperative medicine, Anaesthesia, Surgery

## Abstract

This editorial welcomes the decision of *BJA Open* to publish quality improvement (QI) studies. It summarises the current problems with conducting, evaluating, and publishing QI studies. It highlights existing guidance for prospective authors to follow regarding the reporting of QI interventions, their context(s), underlying theories, and evaluation. In so doing, we hope to encourage the publication of more QI studies of sufficient quality to facilitate learning or replication elsewhere.

We are delighted that *BJA Open* is aiming to provide a vehicle for publishing quality improvement (QI) studies. The authors of the published abstracts from the QI poster presentations at the *Anaesthesia 2022 Conference*^1^ should be applauded for addressing a wide range of quality issues throughout the perioperative pathway. However, the limitations of this format of reporting necessarily constrain the degree of learning which can be communicated. Publishing full QI reports, or evaluations of QI interventions, is a welcome addition to enable readers to learn whether such interventions *could* or *should* be replicated in their setting.

Quality improvement describes a systematic use of repeated measurement and iterative change with the intention of improving a specific aspect of a service.^2^ The analytical methods most commonly used stem from work by W. Edwards Deming,^27^ who developed statistical approaches to characterising variation of processes within the automotive industry. The reliability of clinical systems is poor, leading to delays, wasted effort, frustration, or even harm.^3^ By promoting a ‘bottom-up’ approach to addressing these challenges, QI aims to improve not just patient outcomes but also the wider healthcare system and the working lives of those who deliver care. These aims are particularly pertinent in the UK where the NHS is facing record backlogs in elective surgery alongside severe workforce shortages.^4^^,^^5^

Despite these laudable ambitions, the evidence for impact of QI is mixed.^6^ Partly, this is because poor fidelity to QI methods results in dilution, distortion, or diminution of interventions. Partly, it is a result of multiple short-term small-scale projects failing to be completed or sustained.^7^ A lack of attention to theory or context can also mean that an intervention that succeeds in one setting might fail in another.^8^ Local expertise with QI techniques is unlikely to be sufficient to generate impact without the deployment of socio-organisational functional and facilitative task skills to overcome cultural, organisational, and professional barriers to change.^9^ Reliably demonstrating impact of QI is difficult because typically its evaluation has lacked rigour, relying upon a before-and-after study design, which is vulnerable to bias.^10^ And crucially, there is a widespread failure to aggregate and share learning, despite calls that ‘no improvement intervention should be conducted in isolation’.^11^ For example, synthesising knowledge of QI *via* systematic reviews has been reported to be frustrating because of inconsistent, vague, and variable application of terminology across disciplines.^12^ This failure to share learning effectively is not only a form of research waste but also misses the opportunity for other healthcare providers to decide whether the findings of a QI project could apply to their situation.^13^

Publishing QI studies is therefore a potentially useful method to improve the sharing of findings, but the literature shows that the completeness and quality of such reporting, including in perioperative care, are poor.^14^, ^15^, ^16^ QI studies typically fall into two groups: *QI projects*, which seek to address a local problem, or *evaluation studies*, which seek to generate wider knowledge and can be termed *improvement science*.^15^^,^^17^ QI projects often do not fit the norms of traditional biomedical academic publishing, as they are heterogenous, using broad scopes of both interventions (such as care bundles or reminder systems) and methodologies (such as plan–do–study–act or lean).^18^^,^^19^ Standardising the reporting of such adaptive and iterative projects is challenging, and, as a result, QI projects have often gone unpublished, particularly when their authors have limited time to write up such work alongside clinical responsibilities.^19^ Like other forms of research, there is strong evidence of reporting bias, with ‘positive’ studies much more likely to be reported than ‘negative’ ones.^20^ When reports succeed in being published, they often omit crucial details of the intervention, QI method, context, or unintended consequences, therefore inhibiting replication.^15^^,^^19^^,^^21^^,^^22^

Fortunately, guidance exists on what constitutes a ‘good’ QI report. The Template for Intervention Description and Replication (TIDieR) checklist was published in 2014 to improve reporting of interventions (Table 1).^23^ TIDieR is recommended by the Enhancing the Quality and Transparency of Health Research network as an extension of the Consolidated Standards of Reporting Trials statement.^24^^,^^25^ It provides a valuable guide to describing interventions in sufficient detail to be replicated by others if they so choose.

**Table 1:** Template for Intervention Description and Replication checklist for describing interventions.^23^

Item	Description
Brief name	Provide the name or phrase that describes the intervention
Why	Describe any rationale, theory, or goal of the elements essential to the intervention
What	*Materials:* describe any physical or informational materials used in the intervention, including those provided to participants or used in intervention delivery or in training of intervention providers; provide information on where the materials can be accessed (such as online appendix and URL) *Procedures:* describe each of the procedures, activities, and processes used in the intervention, including any enabling or support activities
Who provided	For each category of intervention provider (such as psychologist and nursing assistant), describe their expertise, background, and any specific training given
How	Describe the modes of delivery (such as face to face or by some other mechanism, such as internet or telephone) of the intervention and whether it was provided individually or in a group
Where	Describe the type(s) of location(s) where the intervention occurred, including any necessary infrastructure or relevant features
When and how much	Describe the number of times the intervention was delivered and over what period of time, including the number of sessions; their schedule; and their duration, intensity, or dose
Tailoring	If the intervention was planned to be personalised, titrated, or adapted, then describe what, why, when, and how
Modifications	If the intervention was modified during the course of the study, describe the changes (what, why, when, and how)
How well	*Planned:* if intervention adherence or fidelity was assessed, describe how and by whom, and if any strategies were used to maintain or improve fidelity, describe them *Actual:* if intervention adherence or fidelity was assessed, describe the extent to which the intervention was delivered as planned

The Standards for Quality Improvement Reporting Excellence 2.0 were published in 2015.^26^ These guidelines are intended for reports that describe and evaluate QI projects and go beyond TIDieR to describe some extremely useful aspects of QI reports that we urge prospective authors to attend to. Firstly, descriptions of the *context* of interventions, and how context influenced the implementation and impact of those interventions, is crucial. Without such information, it is hard for future improvers to decide whether the reported intervention might be successfully implemented in their setting. Secondly, authors should include a consideration of theory (i.e. how and why an intervention might cause the predicted impacts). Third, a holistic appreciation of impacts, including those which are unanticipated, applies to the wider system or represents opportunity costs of engaging with the intervention. Finally, reports should include explicit descriptions of evaluative and analytical techniques, including the use of qualitative techniques alongside quantitative ones.

Improving reporting of QI could make better use of the opportunities provided by increasingly useful perioperative quality infrastructure. For example, several national programmes now support QI by collecting continuous high-quality data sets, setting improvement priorities, and providing QI educational resources. In the UK, the National Emergency Laparotomy Audit and National Hip Fracture Database have been successful perioperative national clinical audits in encouraging reporting of local QI.^20^ It is welcoming to see a number of the abstracts from the *Anaesthesia 2022 Conference*^1^ using Perioperative Quality Improvement Programme (PQIP) data to target national improvement topics, such as individualised preoperative risk assessment, postoperative pain, and encouragement of early drinking, eating, and mobilising after major surgery. Extensive QI resources and tools are also freely accessible on the PQIP website (and on those of some other programmes, such as the Vascular Services Quality Improvement Programme), which can help clinicians with the design, implementation, evaluation, and reporting of QI. The creation of these national QI programmes may therefore help overcome the well-documented weaknesses of short-term, highly local, non-sustained artisanal nature of QI projects.^7^

In conclusion, QI has the capacity to generate significant benefits for patients, providers, and the public, but it is hard to do well. Sharing learning from both ‘positive’ and ‘negative’ studies in accessible and structured formats may help increase the chances of success of future projects. We therefore welcome *BJA Open*'s decision to bring peer-reviewed QI studies to their readership and look forward to reading and learning from the publication of such studies.

## Authors’ contributions

Project conceptualisation/design: DW, SRM.

Drafting of paper: DW.

Review, revision, and approval of paper: both authors.
